# Biology-aware mutation-based deep learning for outcome prediction of cancer immunotherapy with immune checkpoint inhibitors

**DOI:** 10.1038/s41698-023-00468-8

**Published:** 2023-11-06

**Authors:** Junyan Liu, Md Tauhidul Islam, Shengtian Sang, Liang Qiu, Lei Xing

**Affiliations:** Department of Radiation Oncology, Stanford University, Stanford, CA 94305 USA

**Keywords:** Computational biology and bioinformatics, Cancer

## Abstract

The response rate of cancer immune checkpoint inhibitors (ICI) varies among patients, making it challenging to pre-determine whether a particular patient will respond to immunotherapy. While gene mutation is critical to the treatment outcome, a framework capable of explicitly incorporating biology knowledge has yet to be established. Here we aim to propose and validate a mutation-based deep learning model for survival analysis on 1571 patients treated with ICI. Our model achieves an average concordance index of 0.59 ± 0.13 across nine types of cancer, compared to the gold standard Cox-PH model (0.52 ± 0.10). The “black box” nature of deep learning is a major concern in healthcare field. This model’s interpretability, which results from incorporating the gene pathways and protein interaction (i.e., biology-aware) rather than relying on a ‘black box’ approach, helps patient stratification and provides insight into novel gene biomarkers, advancing our understanding of ICI treatment.

## Introduction

Cancer immunotherapy, an approach to utilize the patient’s own immune system against cancer, is recently considered the ‘fourth pillar’ of oncology, besides surgery, radiation therapy, and chemotherapy^1^. Among different types of immunotherapy, immune checkpoint inhibitor (ICI) has revolutionized cancer treatment and provides comprehensive insights into the tumor microenvironment^2^. Cancer cells evade their host’s immune system by negatively regulating T cells via immune checkpoints (e.g., CTLA-4 and PD-1). By blocking these checkpoint proteins, the ability of the immune system to recognize and kill cancer cells restores^3^. Following the authorization of the first ICI drug, ipilimumab, which targets CTLA-4^4^, in 2011, the U.S. Food and Drug Administration (FDA) has since granted clinical approval for various ICI drugs such as anti-PD1 drug pembrolizumab, as well as anti-PDL1 drug atezolizumab^5^. These advancements have significantly broadened the range of effective treatments for numerous types of cancer, including melanoma, non-small-cell lung cancer (NSCLC), and renal cell carcinoma (RCC)^6^. Despite the promising results^7,8^, the individual response rate of ICI varies among patients, with 50–80% in specific types of cancer such as melanoma and Hodgkin lymphoma, while only 15–30% in most other tumors^9^. While studies^9^ have demonstrated the significant impact of tumor mutation burden^10^ on treatment outcomes, a comprehensive understanding of individual gene mutations remains elusive.

**Fig. 1:**
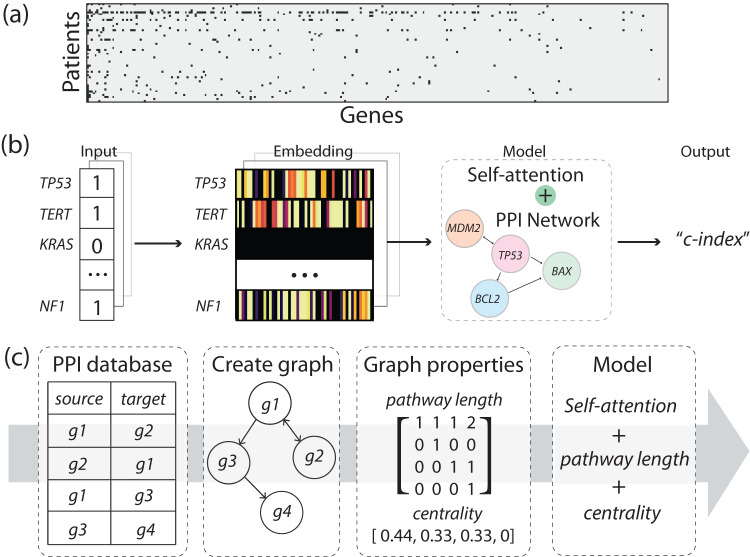
Framework. **a** The diagram in **a** illustrates the sparse and binary nature of mutation data, where each row corresponds to a patient and each column represents a gene. Black dots represent gene mutations, while white space indicates the absence of gene mutations. **b** outlines the framework of our biology-aware model. The mutation data, after undergoing the embedding layer, are fed into the model. The model then generates a predictive score, indicating whether the patient responds to the treatment. Model prediction accuracy is evaluated using the concordance index (c-index). **c** provides a visualization of how we incorporate graph properties into the model. Initially, we construct the graph representation by utilizing information obtained from a publicly accessible protein–protein interaction (PPI) database. Each gene is represented as a node, while the interactions between genes are represented as edges. Subsequently, we calculate various graph properties, such as the shortest pathway length between genes and the centrality of nodes. These properties are utilized to adjust the weight of the self-attention mechanism in the model.

To address this question, survival analysis, including the gold standard Cox regression model, utilizes statistical methods to establish the relationship between treatment response and risk factors (i.e., gene mutations in our application). While the Cox model examines the linear relationships between risk factors, recent advancements in Cox-based deep learning models, including DeepSurv^11^, AECOX^12^, and SurvivalNet^13^, have expanded to investigate the non-linear relationships between risk factors. These models were originally developed for analyzing clinical (e.g., age, sex) and gene expression data. And they are not designed specifically to handle gene mutation data, which consists of solely binary values (where a value of ‘1’ indicates the presence of a mutation and ‘0’ indicates no mutation for each gene, as shown in Fig. 1a). Binary features contain limited information as they represent only two states. Furthermore, they are not suitable for capturing gene relationships and fail to encode the hierarchical structure of gene pathways. None of these models are informed with biological knowledge, despite the wealth of research on genes and pathways that have emerged since the introduction of the Cox model nearly 50 years ago. We hypothesize that a deep learning model that recapitulates the gene pathways and protein interactions may offer new insights into survival analysis. Among deep learning techniques, the self-attention mechanism^14^ holds promise for developing interpretable models, where important genetic biomarkers receive more ‘attention’ (i.e., assigned larger weights). Ying et al. introduced the Graphomer network which leverages both graph and the self-attention to encode structural information^15^. Building upon these previous efforts, we propose a biology-aware mutation-based framework for ICI survival analysis that makes the following contributions: (i) explicitly incorporate gene pathways and protein interactions into the deep learning model, (ii) make interpretable survival predictions, and (iii) identify potential biomarkers.

In our approach, patient mutation data involving 296 genes are transformed into binary arrays (where ‘1’ indicates mutation and ‘0’ represents wildtype genes). As illustrated in Fig. 1b, these arrays undergo an embedding layer, inspired by the concept of semantic relations in word embeddings used in natural language processing (NLP)^16^. Each gene is embedded via a vector representation, and genes with similar expression and co-occurrence are embedded closer to each other in Euclidean space. Afterwards, the embedded data is fed into our model, which incorporates *prior* biology knowledge through the construction of a graph representation. This representation is generated using gene pathways sourced from a publicly available protein–protein interaction (PPI) database^17^. In this graph, individual genes are represented as nodes, while the interactions between genes are represented as edges, as illustrated in Fig. 1c. The impact of mutations along the pathway is assessed based on the graph’s characteristics, including the length of gene pathways and the centrality of the nodes. Genes that are closely connected to mutated genes or have a higher number of interactions in the graph are assigned larger weights. This method mimics the underlying biology where upstream mutations affect the downstream genes. Genes that are directly linked to the mutation receive high attention (i.e., larger weights) as they are directly impacted, while genes further away from the mutations in the graph receive low attention. Eventually, the model outputs a predictive score indicating the patient’s survival outcome. Model accuracy is evaluated via the concordance index (also known as the c-index)^18^, a common statistical method for analyzing censored survival data. Our results demonstrate that the proposed framework achieves accurate and robust predictions across nine types of cancer, outperforming biology-unaware models. The model presents a promising approach to analyze gene mutation data, generate personalized predictions and discover key biomarkers to guide the clinical management of ICI treatment.

## Results

### Biology-aware self-attention mechanism helps identify novel biomarkers

**Fig. 2:**
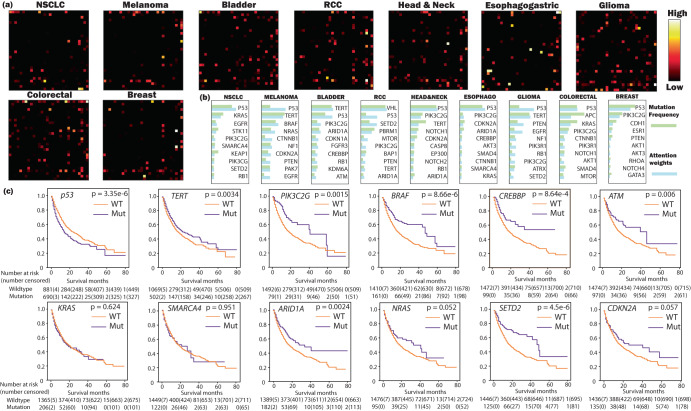
Explore important biomarkers. **a** Displays the average weighted attention map for each type of cancer. Each map, comprising the dimension of 296 by 296 (corresponding to gene number by gene number), has been downsampled to a size of 30 by 30 for illustration purposes (please refer to Supplementary Method 2 for the original attention map). The value at each position (*i*,*j*) signifies the model-assigned attention between gene *i* and *j* by the model. **b** Enumerates the top 10 genes receiving the highest attention. The mutation frequency is plotted in green, while the attention weight is exhibited in blue. Note that a high mutation frequency does not necessarily equal a larger weight. For instance, *P53, in comparison to TERT, BRAF*, and *NRAS*, is less frequently mutated in melanoma patients, yet the model still allocates the highest weight to *P53*. **c** We provide the Kaplan–Meier curves based on the status (mutation vs wildtype) of the genes listed in (**b**). The mutation status of certain genes, including *p53*, *TERT*, *PIK3C2G*, divide patients into groups with significantly different survival status (*p* < 0.1). While some genes (e.g., *KRAS* and *SMARCA4*) do not lead to significant survival differences, their high weights originate from the interactions with other biomarkers.

Interpreting deep learning models poses a significant challenge, particularly within the domain of healthcare^19^. Traditional ‘black box’ deep learning models only allow for reliability assessment based on predictive accuracy, providing little understanding of the model’s decision-making rationale. However, our model leverages the self-attention mechanism, facilitating visualization of the prediction. It achieves this by revealing the attention weights allocated to each gene interaction, thereby introducing additional model validation. (i.e., important genes should receive high attention). Figure 2a illustrates the average weighted attention across patients for each cancer type. The weighted attention (i.e., based on the weighting scheme as described in the “Methods” subsection “Biology-aware self-attention model” has a dimension of 296 by 296 (i.e., gene number by gene number). Thus, we can interpret every entry (*i*, *j*) (*i* ≠ *j*) as the importance of interactions between gene *i* and *j* towards the treatment outcome, and interpret the diagonal entry (*i*, *i*) as the importance of the specific gene *i*. This explanation motivates our investigation into biomarkers by selecting the genes with the highest attention. We then identify the top ten highest-attention genes, as demonstrated in Fig. 2b. Our model represents a different approach compared to the original COX-PH model. The original Cox-PH model, described in Eq. (1), determines risk hazard *h* according to the base hazard *h*_*0*_ and gene mutation *x*_*i*_. If the gene mutates, *x*_*i*_ = 1; otherwise, it is 0. The learnable parameter *β*_*i*_ represents the weights of individual gene. However, our model’s weights are based on gene interaction pairs. Genes without interaction receive low attention (as denoted by black in Fig. 2a) while critical interactions receive high attention (as denoted by yellow and white in Fig. 2a). Note that it is feasible to include the interaction term into the COX-PH model (e.g., by adding the *x*_*i*_*x*_*j*_ product term). By leveraging a deep learning framework, we are relieved of the need to manually define the interaction term. This is particularly beneficial when dealing with potentially complex, non-linear gene interaction, which can be arduous to define explicitly.

1 $$h={h}_{0}\cdot \exp \left(\sum {\beta }_{i}{x}_{i}\right)$$

To validate our findings, we initiated a comprehensive literature review of all the listed genes. The literature review is provided in Supplementary Method 1.

In addition to the literature review, we present the pan-cancer survival analysis by providing the Kaplan–Meier curves of patients categorized by the status (i.e., mutated or wildtype) of the identified genes, as depicted in Fig. 2c. It is important to mention that not all identified genes can stratify patients into two groups with a significant survival difference (*P* > 0.1, e.g., *KRAS*, *p* = 0.624; *SMARCA4*, *p* = 0.951). Nonetheless, these genes are linked to biomarkers that do stratify patients with substantial survival disparities. (e.g., *KRAS* is an upper stream gene of *BRAF*, *p* = 8.66e−6 < 0.1; *SMARCA4* interacts with *ARID1A*, *p* = 0.0024 < 0.1). When we calculate the attention of individual genes (as described in the “Methods” subsection “Biomarker discovery and validation”, these biomarkers receive considerable weight due to their interactions with other high-attention genes.

### The predictive scores correlate with the TMB and MSI status

**Fig. 3:**
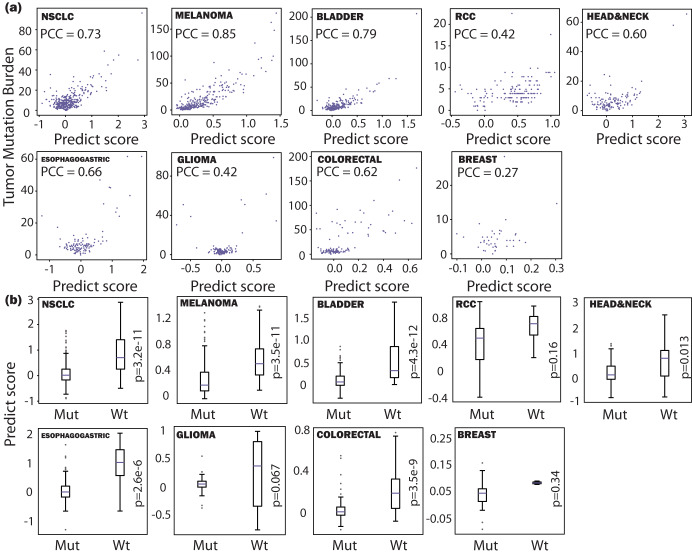
Model prediction analysis. Compare model predictive scores to **a** Tumor mutation burden (TMB) and **b** Mutations in MMR genes. TMB values are directly available for the same dataset. The status of dMMR is inferred from mutations in MMR genes, which include the mutation in *MLH1*, *MSH2*, *MSH3*, *MSH6*, and *PMS2* genes. Patients with or without these mutations are labeled “Mut” and “Wt”, respectively. Note that the mutations in MMR genes do not perfectly align with the clinical dMMR status, and this primarily serves as a “sanity check” of the model predictions due to this limitation.

Tumor mutation burden (TMB) is recognized as a predictive biomarker of the ICI treatment response^20,21^, Similarly, a deficiency in mismatch repair genes (dMMR), potentially resulting in elevated levels of microsatellite instability (MSI), has also been associated with the response rates to ICIs^22,23^, Although our model does not explicitly incorporate TMB or dMMR status as part of its inputs, we conduct an examination whether our model’s predictive scores align with these well-established indicators. Figure 3a and b respectively portray the results of the comparison between TMB and MSI status against predictive scores. The TMB values are directly available for the same dataset. The MSI status is inferred from the mutation status of the Mismatch Repair (MMR) genes, which includes the mutations of the *MLH1*, *MSH2*, *MSH3*, *MSH6*, and *PMS2*^24^. If patients exhibit no mutations in these MMR genes, they are classified as “Wt” in Fig. 3. If such mutations are present, they are designated as “Mut” in Fig. 3. Figure 3b is used as an additional “model sanity check”, as patients with these mutations are likely to have dMMR tumors. However, note that the mutations in MMR genes do not perfectly align with the clinical definition of dMMR/ MSI, which is a limitation of the current approach. (e.g., the most common cause for dMMR in colorectal cancer is *MLH1* promoter hypermethylation^25^, but this information is not available in the current dataset).

As a result, the predictive scores demonstrate a strong correlation with TMB and indicate a statistically significant survival disparity between patients with mutations in MMR genes (*p* < 0.05). Only three cancer types are exceptions to this trend. In the cases of RCC and glioma, our model exhibits relatively low predictive accuracy, indicating potential prediction errors. Regarding breast cancer, the ability to draw robust statistical conclusions is constrained by the limited sample size of only 44 patients present in the dataset.

### Better prediction accuracy compared to conventional survival analysis

**Fig. 4:**
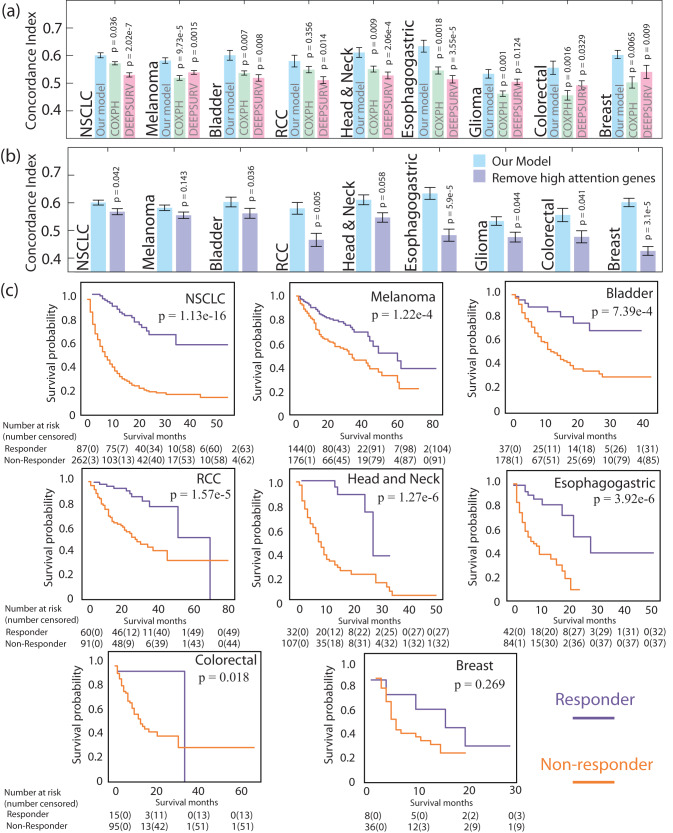
Model prediction. **a** Shows the averaged prediction accuracy (via c-index) for each cancer type (mean ± standard error), compared to Cox-PH and DeepSurv models. *P* values of Cox-PH and DeepSurv are computed via ANOVA test against our model. **b** We remove high-attention genes (i.e., genes listed in Fig. 2b) for each type of cancer, re-train and re-predict the model, and observe a decrease in prediction accuracy (mean ± standard error). *P* values are computed on prediction accuracy with or without high attention genes. **c** illustrates the patient stratification into responders and non-responders based on the model predictive score.

We train, validate, and test the model on 1571 patients across nine types of cancer. The prediction accuracy (i.e., c-index) is derived from an average of repeated ten-fold cross-validation (as described in the “Methods” subsection “Model training, validation and testing”. We achieve an averaged c-index of 0.601 ± 0.009 (mean ± standard error) for NSCLC, 0.582 ± 0.010 for melanoma, 0.603 ± 0.018 for bladder cancer, 0.580 ± 0.021 for RCC, 0.611 ± 0.018 for head and neck cancer, 0.634 ± 0.022 for esophagogastric cancer, 0.534 ± 0.016 for glioma, 0.556 ± 0.024 for colorectal cancer, and 0.601 ± 0.015 for breast cancer, respectively, as illustrated in Fig. 4a. See Supplementary Method 4 for the fitting outcome of *k*_1_, *k*_2_ and *k*_3_. Our model significantly outperforms the commonly used Cox-PH model and DeepSurv (*p* < 0.05 in all cancer types via one-way ANOVA test except two, detailed below). Note that DeepSurv was originally developed for tabular clinical data, not gene mutation data. However, it remains relevant as it represents one of the simplest non-linear models. Our prediction shows an insignificant accuracy increase (*p* > 0.05) for two cancer types: glioma and RCC. For glioma, nearly 90% of patients in our dataset die within the first 30 months, posing a challenge to discern the effectiveness of treatment. Studies have suggested^26,27^, the ICI treatment does not show consistent survival improvement for glioma patients. Low ICI response rates contribute to the insignificant performance compared to DeepSurv (*p* = 0.124). As for RCC, *VHL* is listed as the highest-attention gene (as seen in Fig. 2b); however, even though *VHL* is a recognized biomarker for RCC^28^, we find insufficient evidence in the current literature regarding its role in immunotherapy. This suggests a potential error in attention, resulting in the model’s poor accuracy compared to Cox-PH (*p* = 0.356).

Subsequently, we remove the high-attention genes from the data (i.e., remove genes listed in Fig. 2b), re-train and re-predict the model. We observe a notable decrease (*p* < 0.1) in the overall prediction accuracy, as depicted in Fig. 4b, confirming the predictive value of these biomarkers for the treatment outcome. Nonetheless, for melanoma, we observe an insignificant accuracy reduction (*p* > 0.1). Upon examining the new attention map post the removal of high-attention genes, we discern that the model now places more attention on genes *HRAS*, *AKT2*, *PIK3CA* for melanoma. These genes interact closely with the removed genes (e.g., within the *PI3K-PTEN-AKT* pathway^29^). These observations suggest that, even in the absence of certain gene biomarkers in the data, the model may identify alternative upstream or downstream genes during the prediction process. This explains why the removal of high-attention genes does not significantly impair the prediction accuracy.

### Patient stratification based on the predictive scores

In this study, patient stratification refers to classifying patients into distinct groups—non-responders and responders—based on the predictive scores generated by our model. We have excluded glioma from this process due to its low response rates and significant variability across different studies. When a new patient presents with known mutations, we can run the model with calibrated weights and categorize the patient as either a responder or a non-responder. This technique could potentially contribute to the personalization of ICI treatment, tailored to the patient’s gene mutation profile. The Kaplan–Meier survival curves for both subgroups are illustrated in Fig. 4c. The curve positioned lower, representing a shorter survival duration, corresponds to non-responders, whereas the curve positioned higher, indicative of extended survival, pertains to responders.

## Discussion

In this paper, we introduce a biology-aware mutation-based model for predicting the ICI treatment outcome. We then validate the model on nine types of cancer. Previous studies have explored gene expression and clinical tabular data (e.g., age, sex) using various learning-based survival models^11–13^ While gene mutation is another common measurement, it is rarely incorporated into such models since it consists solely of binary values, which conveys limited information and are not suitable to represent gene relationships. A preliminary attempt was made by the clinical transformer^30^, which directly applied the transformer structure to mutation data and achieved accurate predictions. However, the model still implicitly learns the gene interactions from the data, without considering *prior* biological knowledge. This study addresses this gap by presenting a method to combine the binary mutation data with deep-learning survival models.

The proposed method offers several advantages compared to previous survival models on mutation data. Genes are embedded into continuous vectors based on their expression. The weighted attention map mimics the underlying biology where an upstream gene mutation can affect the downstream. Additionally, we validate our model prediction based on the identified biomarkers. Indeed, we have performed a literature review and compared the model predictions with TMB and MSI statuses. Several identified biomarkers (e.g., *P53*, *KRAS*, *BRAF*) align with previous studies^31^ that already used mutations as predictors of ICI treatment response. This method has the potential to advance our understanding of current ICI treatment and reduces the risk of drawing unreliable conclusions from ‘black boxes’ deep learning models that are difficult to interpret.

Several factors may affect the model’s accuracy. Primarily, our current model solely considers gene mutation data, excluding patient clinical information such as age and sex. While this enables the model to concentrate exclusively on mutation biomarkers, we may also overlook valuable factors. For example, the human immune system is known to be influenced by age^32^, a variable not currently accounted for in our model. Meanwhile, gene expression provides additional information beyond mutation data. Incorporating other clinical data (e.g., age, gene expression) into our model is a future direction we are actively pursuing. Secondly, our approach makes several simplifications. Our PPI graph only accounts for the direct or indirect interactions among the 296 genes present in our data. Biologically, it’s possible for two genes to be indirectly linked via a third gene not included in our current selection of 296 genes. Nevertheless, considering all genes in the PPI library would significantly increase the computational cost associated with constructing the PPI graph. Our model also simplifies the mutation status as binary, neglecting the variety of mutation types, including deletions, missense mutations, and frameshift mutations. Lastly, survival outcomes (i.e., how long a patient survives following treatment) result from complex factors such as the tumor microenvironment, pre-treatment health status, pharmacodynamics, and others, in addition to gene mutation. Like all other survival models, ours does not encapsulate every aspect of ICI treatment and, as such, has its limitations.

In this study, we developed a biology-aware mutation-based deep learning model integrating gene pathways and protein interactions. This framework enables interpretable survival analysis for patients receiving ICI treatment, highlighting its capacity to uncover predictive biomarkers. The model is not limited to immunotherapy and can be extended to other types of cancer treatment (e.g., targeted therapy) where gene mutation is crucial, thereby having an impact beyond ICI treatment. For future studies, we plan to incorporate additional clinical information into the framework such as the multivariate survival analysis and the MSI score. It is also crucial to examine whether this learning-based approach adds value beyond conventional biomarkers in patient care.

## Methods

### Dataset ethics and preprocessing

Our study utilized a dataset comprising 1571 patients across nine cancer types treated with ICI at Memorial Sloan Kettering Cancer Center (MSKCC), collected via the MSK-IMPACT assay^33^. This includes 349 NSCLC patients, 320 melanoma patients, 215 bladder cancer patients, 151 RCC patients, 139 head and neck cancer patients, 126 esophagogastric cancer patients, 117 glioma patients, 110 colorectal cancer patients, and 44 breast cancer patients. Note that we remove patients whose cancer types are labeled unknown^34^. The protocols of sequencing and variant calling are available in the original study^35^. This dataset is institutionally reviewed and approved by MSKCC and is downloaded from the open-source database cBioPortal^36^. For each patient, mutation data involving 468 genes and clinical information are available. The survival status and the overall survival serve as our ‘ground truth’ for model training and prediction. During data preprocessing, genes mutated in <1% of the total population (i.e., genes that mutate in ≤16 patients) were removed, reducing the gene count to 296. Each patient is represented as an array consisting of binary values, indicating whether the gene is mutated (i.e., ‘1’ for mutation and ‘0’ for wildtype).

### Embedding layer

**Fig. 5:**
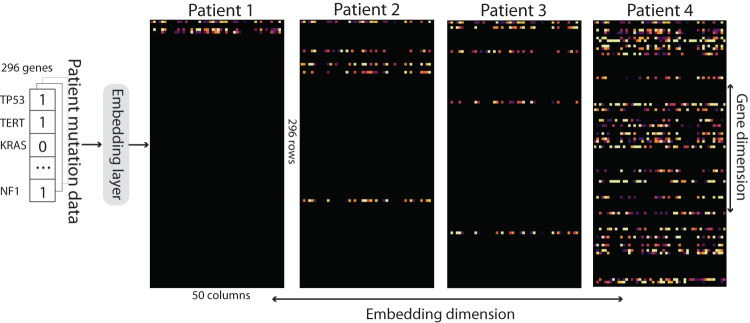
Embedding layer. This figure shows the embedding outcomes for four sample patients. Their respective mutation data, initially encoded as binary vectors of dimension 296 by 1, are transformed into a 296 by 50 matrix. In this format, each gene is signified by a 50-dimensional array, embodying information about gene expression and co-occurrence derived from TCGA data. For each patient, the rows correspond to genes (totaling 296 rows), and the columns represent the associated embeddings.

To address the data non-continuity resulting from the binary nature of the mutation input, we represent each gene with a 50-dimensional vector via a previous embedding method proposed by Choy et al.^37^. These embeddings are achieved through a shallow two-layer artificial neural network trained on transcriptomic data from The Cancer Genome Atlas (TCGA), encapsulating gene expression and co-occurrence. Once generated, these embeddings remain fixed throughout the study. Consequently, each patient is characterized by a 296-by-50 matrix, where mutated genes are substituted with their corresponding embedding vectors, and wildtype genes, originally marked as ‘0’, are represented by a 50-dimensional zero vector, as shown in Fig. 5. The potential influence of the embedding dimension is examined further in Supplementary Method 3.

### Biology-aware self-attention model

To make the model biology-aware, we create a graph based on the protein–protein interaction (PPI) database sourced from the UCSC genome browser^17^. This database captures interactions of human genes that have been identified through biological experiments or PubMed text mining. By targeting the 296 genes of interest, we extract a total of 6004 gene interactions. Utilizing the Python library ‘networkx’, we establish our PPI graph by setting the 296 genes as nodes and the 6004 gene pairs as graph edges. This is a directed graph as the library specifies the direction of interactions (gene A pointing to B means A acting upon B). The function of PPI graph is analogous to the “positional encoding” for words in the natural language processing. The whole process is illustrated in Fig. 1c.

**Fig. 6:**
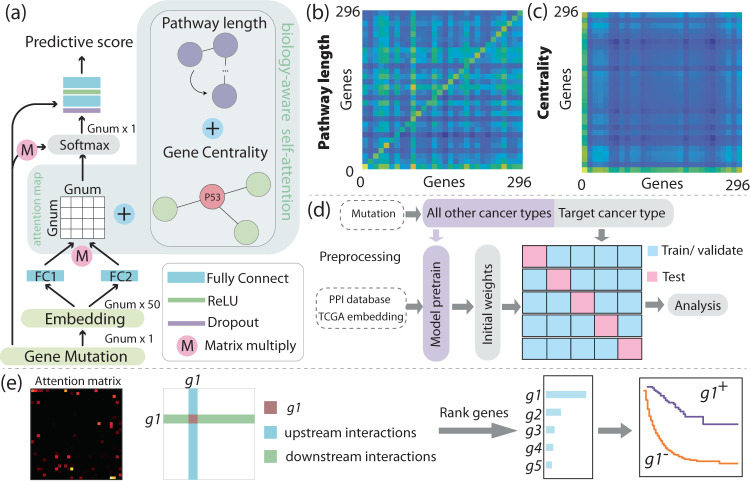
Model structure and attention calculation. **a** The biology-aware model with attention weighted by **b** pathway matrix, **c** connection matrix, and padding matrix. The pathway matrix characterizes the reciprocal of the shortest path from gene *i* to *j*. In **b**, this matrix is downsampled to 30 by 30 for illustrative convenience. The genes that are directly connected to mutated genes will receive high attention compared to distant genes. **c** quantifies the centrality. Mutated nodes with larger centrality values can potentially affect a more extensive set of genes compared to those with lower centrality. **d** iIllustrates the model training, validation, and testing procedure via repeated cross-validation. **e** Details of the process of identifying biomarkers, beginning with the computation of individual gene weights from the attention map, followed by ranking and discerning the top 10 genes with the highest attention. Kaplan–Meier curves are subsequently plotted based on gene mutation status.

Following the embedding layer, each patient is represented by a 296 by 50 matrix denoted as *x*. The attention map is acquired with Eq. (2), where FC1 and FC2 represent the two fully connected layers illustrated in Fig. 6a.

2 $$\begin{array}{ll}{\rm{attention}}\,{\rm{map}}=\frac{{\rm {F{C}}}_{1}(x)\cdot {\rm {F{C}}}_{2}{(x)}^{{\rm {T}}}}{\sqrt{{d}_{k}}}+{k}_{1}\cdot {\rm {pathway}}\\ \qquad\qquad\qquad\quad+\,{k}_{2}\cdot {\rm {connection}}\\ \qquad\qquad\qquad\quad+\,{k}_{3}\cdot {\rm {padding}}\end{array}$$

The first term on the right-hand side is the biology-unaware attention score. It is calculated by multiplying the output matrices produced by two fully connected layers and then normalizing by the embedding dimension *d*_*k*_ = 50. This attention score is subsequently weighted against three additional matrices: the pathway matrix, the connection matrix, and the padding matrix. All four matrices (including the attention map) have a shape of 296 by 296. *k*_1_, *k*_2_, and *k*_3_ are learnable parameters that determine the relative weights among these matrices. Without these additional matrices, the attention map only reflects the implicit relationships learned solely from the data. The overall goal of this step is to adjust the weights of a given gene interaction (*i*, *j*) based on our *prior* biological knowledge.

The ‘pathway’ matrix assigns greater weights to closely associated genes. Specifically, it quantifies the shortest path length from gene *i* to gene *j*, as per Eq. (3). If gene *i* and *j* are disconnected on the graph, the value at (*i*, *j*) is 0; if connected, it is the reciprocal of the shortest path length *L*_shortest_ navigating from gene *i* to *j*. The ‘pathway’ matrix is shown in Fig. 6b.

3 $${\rm {pathway}}(i,j)=\left\{\begin{array}{ll}0, & {\rm{if}}\,i,j\,{\rm{not}}\,{\rm{connected}}\\ \frac{1}{{L}_{{\rm {shortest}}}}, & {\rm{if}}\,{\rm{shortest}}\,{\rm{path}}\,{\rm{from}}\,i\,{\rm{to}}\,j\,{\rm{exists}}\end{array}\right.$$

The ‘connection’ matrix quantifies the closeness centrality of gene pair (*i*, *j*) based on Eq. (4). In graph theory, centrality measures the importance of nodes. The mutation that connects to a significant number of downstream genes warrants larger weights over genes with fewer connections. The degree of centrality is shown in Fig. 6c.

4 $${\rm {connection}}(i,j)={\rm {centralit{y}}}_{i}+{\rm {centralit{y}}}_{j}$$

The ‘padding’ matrix serves as an additional penalty for gene pairs (*i*, *j*) that are not connected (i.e., mutation of gene *i* will not affect *j*). This penalty can be mathematically described as Eq. (5).

5 $${\rm{padding}}(i,j)=\left\{\begin{array}{ll}0, & {\rm{if}}\,i,j\,{\rm{are}}\,{\rm{connected}}\\ -1, & {\rm{if}}\,i,j\,{\rm{are}}\,{\rm{not}}\,{\rm{connected}}\end{array}\right.$$

### Loss function for survival analysis

The concordance index (c-index) is one of the statistical methods to evaluate the model accuracy with censored data^18^ by comparing the prediction for every pair of patients *i* and *j*. We name (*i*,*j*) as a concordant pair if the predictive score *p*_*i*_ > *p*_*j*_ when the overall survival *T*_*i*_ > *T*_*j*_, given both patients decrease, or patient *i* is surviving while *j* decreases. Conversely, if both patients are still alive, or *T*_*i*_ > *T*_*j*_ when patient *i* is decreased while *j* survives, we label the pair (*i*,*j*) as incomparable since patient *j* ’s eventual survival duration remains uncertain. The c-index is defined as the proportion of concordant pairs to all comparable pairs^38^. The original concordance index is discrete and does not have a continuous gradient for optimization. As a solution, we implement a sigmoid approximation based on Schmid et al.^39^. Survival models typically offer two prediction types: those estimating patient response efficacy and those providing a ‘risk score’. Our model falls under the first type. Accordingly, we seek to maximize the c-index by minimizing the loss function as Eq. (6). L_2_ regularization defined as the sum of squared model parameters, is utilized as a common strategy to reduce overfitting.

6 $${\rm {loss}}=1-{\rm{cindex}}+0.01\cdot {L}_{2}{\rm {regularization}}$$

### Model training, validation, and testing

In order to fully utilize the available data, we employ a pre-training procedure with repeated 10-fold cross-validation, as depicted in Fig. 6d. Initially, for each cancer type, we pre-train the model using all patient data from the other eight types, excluding the target cancer. Subsequently, for the specified cancer type, the model is initialized with pre-trained weights, and a ten-fold cross-validation is conducted. For each round of cross-validation (i.e., ten rounds in total), the target cancer patients are randomly divided into a 10% test set, used for model accuracy evaluation, and a 90% non-test set, for model parameter calibration, using the scikit-learn “train_test_split” function. This guarantees that the data used for assessment remains unseen by the model during the training and validation. Within the 90% non-test set, the data is further randomly split into an 80% training set and a 20% validation set using “train_test_split”. Model parameters that yield the highest validation accuracy are saved and then used to make predictions on the test sets.

This entire procedure is repeated five times, generating 50 values (i.e., 10-fold multiplied by five iterations). We use the mean and standard error of these 50 values to represent our model accuracy. Notably, for breast cancer, due to the limited patient count of 44, we conducted a repeated five-fold cross-validation instead of 10-fold.

### Numerical implementation

Data preprocessing is performed via Python library Pandas 1.4.1. Deep learning models are developed via Pytorch 1.11.0. All data processing, model calibration and prediction were performed on a computer with a 3.7 GHz Intel i9-10900K and Nvidia 3080 Ti. Using this system, a single ten-fold cross-validation took <30 min with 200 epochs and a learning rate 1e−3. Cox-PH is implemented by Python library ‘lifelines’^40^, while DeepSurv is implemented by Python library ‘Pycox’^41^. Figures are plotted with Python library ‘Matplotlib’ 3.6.3.

### Biomarker discovery and validation

To assess the significance of individual genes, we scrutinize the attention weights associated with each gene, as illustrated in Fig. 6e. We sum up all weights involving a specific gene as Eq. (7), including the downstream interactions (the first term), the upstream interactions (the second term), as well as the self-attention (the third term). In Eq. (7), the weight(*i*) represents the total weights of the specific gene *i*, while att(*i*,*j*) denotes the attention map (*i*,*j*). To ensure a fair comparison between frequently and infrequently mutated genes, we normalize the attention value of each gene by its mutation frequency, which is calculated as the proportion of patients with a specific gene mutation over the total number of patients. We then ranked the top 10 genes with the highest weights.

7 $${\rm {weight}}(i)=\mathop{\sum }\limits_{j=1,j\ne i}^{296}{\rm {att}}(i,j)+\mathop{\sum }\limits_{j=1,j\ne i}^{296}{\rm {att}}(j,i)+{\rm {att}}(i,i)$$

To validate the credibility of the identified biomarkers, we employ three verification procedures: (1) A literature review to ascertain the role of the identified genes in ICI treatment. (2) The retraining, revalidation, and retesting of the model after removing all identified genes from the data, to assess if their exclusion leads to reduced prediction accuracy. (3) Stratification of patients into two groups based on gene mutation and plotting of their Kaplan–Meier survival curves.

### Patient stratification

It is feasible to cluster responders from non-responders based on predictive scores. While the model generates a predictive score indicating the relative survival status, it does not know the clinical threshold to distinguish between responders and non-responders. Documented response rates from existing literature^42^ are used for this purpose (e.g., for instance, research indicates that breast cancer patients have an overall response rate of 17% to ICI treatment. Consequently, we classify patients with the top 17% of predictive scores as responders). Kaplan–Meier curves for each subgroup are then plotted via Python function ‘kaplan_meier_estimator’ from ‘sksurv’. And the *P* values between the two groups are calculated using the function ‘compare_survival’ from ‘sksurv’.

### Reporting summary

Further information on research design is available in the Nature Research Reporting Summary linked to this article.

### Supplementary information

Supplementary Material

REPORTING SUMMARY

## Data Availability

All data used for analysis are accessible from public database cBioPortal (https://www.cbioportal.org/).

## Code availability

The code used for the model is accessible from https://github.com/JunyanReplicant/ICImodel.

## References

[1] McCune JS (2018). Rapid advances in immunotherapy to treat cancer. Clin. Pharmacol. Ther..

[2] Robert, C. A decade of immune-checkpoint inhibitors in cancer therapy. *Nat. Commun*. **11**, Art. no. 1 (2020). 10.1038/s41467-020-17670-y PMC7393098 32732879

[3] Ribas A, Wolchok JD (2018). Cancer Immunotherapy using checkpoint blockade. Science.

[4] Sondak, V. K., Smalley, K. S. M., Kudchadkar, R., Grippon, S. & Kirkpatrick P. Ipilimumab. *Nat. Rev. Drug Discov*. **10**, Art. no. 6 (2011). 10.1038/nrd3463 21629286

[5] Ai L (2020). Research status and outlook of PD-1/PD-L1 inhibitors for cancer therapy. Drug Des. Dev. Ther..

[6] Hargadon KM, Johnson CE, Williams CJ (2018). Immune checkpoint blockade therapy for cancer: an overview of FDA-approved immune checkpoint inhibitors. Int. Immunopharmacol..

[7] Thomas, R., Al-Khadairi, G. & Decock, J. Immune checkpoint inhibitors in triple negative breast cancer treatment: promising future prospects. *Front. Oncol*. **10**, https://www.frontiersin.org/article/10.3389/fonc.2020.600573 (2021). 10.3389/fonc.2020.600573 PMC7947906 33718107

[8] Darvin, P., Toor, S. M., Sasidharan, Nair V. & Elkord, E. Immune checkpoint inhibitors: recent progress and potential biomarkers. *Exp. Mol. Med*. **50**, Art. no. 12, (2018). 10.1038/s12276-018-0191-1 PMC6292890 30546008

[9] Esfahani K (2020). A review of cancer immunotherapy: from the past, to the present, to the future. Curr. Oncol..

[10] Bao R (2014). Review of current methods, applications, and data management for the bioinformatics analysis of whole exome sequencing. Cancer Inform..

[11] Katzman JL (2018). DeepSurv: personalized treatment recommender system using a Cox proportional hazards deep neural network. BMC Med. Res. Methodol..

[12] Huang Z. et al. Deep learning-based cancer survival prognosis from RNA-seq data: approaches and evaluations. *BMC Med. Genom.* **13**, 41 (2020). 10.1186/s12920-020-0686-1 PMC7118823 32241264

[13] Yousefi, S. et al. Predicting clinical outcomes from large scale cancer genomic profiles with deep survival models. *Sci. Rep*. **7**, Art. no. 1 (2017). 10.1038/s41598-017-11817-6 PMC5601479 28916782

[14] Vaswani, A. et al. Attention is all you need. *Adv. Neural Inf. Process. syst.* **30** (2017)

[15] Ying, C. et al. Do transformers really perform bad for graph representation? Preprint at *arXiv*10.48550/arXiv.2106.05234 (2021).

[16] Mikolov, T., Chen, K., Corrado, G. & Dean J. Efficient estimation of word representations in vector space. Preprint at *arXiv*10.48550/arXiv.1301.3781 (2013).

[17] Poon H, Quirk C, DeZiel C, Heckerman D (2014). Literome: PubMed-scale genomic knowledge base in the cloud. Bioinforma. Oxf. Engl..

[18] Brentnall AR, Cuzick J (2018). Use of the concordance index for predictors of censored survival data. Stat. Methods Med. Res..

[19] Miotto R, Wang F, Wang S, Jiang X, Dudley JT (2018). Deep learning for healthcare: review, opportunities and challenges. Brief. Bioinform..

[20] Chan TA (2019). Development of tumor mutation burden as an immunotherapy biomarker: utility for the oncology clinic. Ann. Oncol..

[21] Burtness B (2020). Correlation of tumor mutational burden (TMB) with CDKN2A and TP53 mutation in HPV-negative head and neck squamous cell carcinoma (HNSCC). J. Clin. Oncol..

[22] Wang Q-X (2021). The degree of microsatellite instability predicts response to PD-1 blockade immunotherapy in mismatch repair-deficient/microsatellite instability-high colorectal cancers. Exp. Hematol. Oncol..

[23] Motta R (2021). Immunotherapy in microsatellite instability metastatic colorectal cancer: current status and future perspectives. J. Clin. Transl. Res..

[24] Li, G.-M. Mechanisms and functions of DNA mismatch repair. *Cell Res*. **18**, Art. no. 1 (2008). 10.1038/cr.2007.115 18157157

[25] Niv Y (2007). Microsatellite instability and MLH1 promoter hypermethylation in colorectal cancer. World J. Gastroenterol..

[26] Xu S, Tang L, Li X, Fan F, Liu Z (2020). Immunotherapy for glioma: current management and future application. Cancer Lett..

[27] Desbaillets N, Hottinger AF (2021). Immunotherapy in glioblastoma: a clinical perspective. Cancers.

[28] Cowey CL, Rathmell WK (2009). VHL gene mutations in renal cell carcinoma: role as a biomarker of disease outcome and drug efficacy. Curr. Oncol. Rep..

[29] Hsin, I.-L., Shen, H.-P., Chang, H.-Y., Ko, J.-L. & Wang, P.-H. Suppression of PI3K/Akt/mTOR/c-Myc/mtp53 positive feedback loop induces cell cycle arrest by dual PI3K/mTOR inhibitor PQR309 in endometrial cancer cell lines. *Cells* **10**, Art. no. 11 (2021). 10.3390/cells10112916 PMC8616154 34831139

[30] Kipkogei, E., Argoty, G. A. A., Kagiampakis, I., Patra, A. & Jacob, E. Explainable transformer-based neural network for the prediction of survival outcomes in non-small cell lung cancer (NSCLC). Preprint at medRxiv 10.1101/2021.10.11.21264761 (2021).

[31] Gajic, Z. Z., Deshpande, A., Legut, M., Imieliński, M. & Sanjana, N. E. Recurrent somatic mutations as predictors of immunotherapy response. *Nat. Commun*. **13**, Art. no. 1 (2022). 10.1038/s41467-022-31055-3 PMC9270330 35803911

[32] Castelo-Branco C, Soveral I (2014). The immune system and aging: a review. Gynecol. Endocrinol..

[33] Zehir A (2017). Mutational landscape of metastatic cancer revealed from prospective clinical sequencing of 10,000 patients. Nat. Med..

[34] Samstein, R. M. et al. Tumor mutational load predicts survival after immunotherapy across multiple cancer types. *Nat. Genet*. **51**, Art. no. 2 (2019). 10.1038/s41588-018-0312-8 PMC6365097 30643254

[35] Cheng DT (2015). Memorial Sloan Kettering-integrated mutation profiling of actionable cancer targets (MSK-IMPACT). J. Mol. Diagn..

[36] Gao J (2013). Integrative analysis of complex cancer genomics and clinical profiles using the cBioPortal. Sci. Signal..

[37] Choy, C. T., Wong, C. H. & Chan, S. L. Embedding of genes using cancer gene expression data: biological relevance and potential application on biomarker discovery. *Front. Genet*. **9**, https://www.frontiersin.org/article/10.3389/fgene.2018.00682 (2019). 10.3389/fgene.2018.00682 PMC6329279 30662451

[38] Steck, H., Krishnapuram, B., Dehing-oberije, C., Lambin, P. & Raykar, V. C. On ranking in survival analysis: bounds on the concordance index. In *Advances in Neural Information Processing Systems*, 20 (Curran Associates, Inc., 2007).

[39] Mayr A, Schmid M (2014). Boosting the concordance index for survival data—a unified framework to derive and evaluate biomarker combinations. PLoS ONE.

[40] Davidson-Pilon C (2019). Lifelines: survival analysis in Python. J. Open Source Softw..

[41] Kvamme H, Borgan Ø, Scheel I (2019). Time-to-event prediction with neural networks and Cox regression. J. Mach. Learn. Res..

[42] Valero C (2021). Response rates to anti-PD-1 immunotherapy in microsatellite-stable solid tumors with 10 or more mutations per megabase. JAMA Oncol..

