# Supplementary material for: Biology-aware mutation-based deep learning for outcome prediction of cancer immunotherapy with immune checkpoint inhibitors
Source: NPJ Precis Oncol. 2023 Nov 6;7:117. doi: 10.1038/s41698-023-00468-8 (PMC10628135; doi:10.1038/s41698-023-00468-8)
Supplement: Supplementary file 1 — Supplementary Material [file 41698_2023_468_MOESM1_ESM.docx]

**Supplementary Material for Biology-Aware Mutation-based Deep Learning for Outcome Prediction of Cancer Immunotherapy with Immune Checkpoint Inhibitors**

^1^Junyan Liu, ^1^Md Tauhidul Islam, ^1^Shengtian Sang, ^1^Liang Qiu, and ^1^Lei Xing

1. Department of Radiation Oncology, Stanford University, Stanford, California, 94305, USA

**1. Literature review of genes with high attention weights.**

Here we list the literature support for all the other genes listed in the Figure 2b. The model identifies *TP53*, often referred to as the ‘guardian of genome’[1], and has been shown to impact tumor mutation burden (TMB) [2]. It also underscores the telomerase reverse transcriptase *TERT*, recently validated through pan-cancer analysis to possess predictive value for ICI treatment [3],[4]. The *PI3K-PTEN-AKT-mTOR* pathway, which involves genes including *PIK3CG*, *PIK3C2G, PIK3R1*, has been associated with the modulation of the tumor microenvironment (TME) and resistance to immunotherapy [5]–[7]. The Notch (1-4) family, linked with T cell development and activation [8], has been shown to regulate the immune response in TME [9]. *CDKN1A*, also known as p21, associates with both the pro-inflammatory reprogramming of macrophages [10] and TMB [11]. Studies have found immune-inflammatory pathways are suppressed in CDKN2A[12] altered tumors. The *CREBBP*[13] mutation is associated with higher tumor mutation burden in pan-cancer analysis. Nonsense mutation of *ATM* [14] is beneficial for the ICI therapy. Alterations in *ARID1A* [15] marks longer progression free survival status after ICI treatment. *RAS* family [16] can enhance PD-L1 expression, thereby improving the ICI treatment outcome. In contrast, mutation in *SMAD4* [17, p. 4] reduces PD-L1 expression, leading to poor ICI response. Pan cancer analysis has shown *SETD2* [18, p. 2] is linked to favorable clinical outcomes. APC [19] corresponds to poor ICI response in colon cancer. PBRM1 [20] mutation correlates with immune response. *Rho* family [21], [22] has multiple functions in tumor microenvironment, such as recruiting immune suppressive cells MDSCs or TAMS, thereby becoming a potential target for ICI treatment. *EP300* [23] inhibits anti-tumor immune response. *SMARCA4* [24] often co-mutates with *KEAP1* [25], and *STK11* [26], and is associated with immunotherapy resistance. *CTNNB1* [27] is related to reduced level of active immune cells. *RB1* [28], [29] has shown immunological features and may improve the ICI treatment sensitivity. *EGFR* [30], [31] mutation is linked to decreased PD-L1 expression and shows poor ICI response in lung cancer patients. *PAK7* [32] is related to higher tumor mutation burden and increased neoantigen load. BRAF in melanoma [33], [34] has been linked to tumor infiltration by T cells. *CDH1* [35] is identified as predictors of resistance to ICIs. *CASP8* [36] inhibits PD-L1 expression. Loss of BAP1 [37] is linked to a more inflamed tumor microenvironment. KDM6A [38] is linked to the immune escape of bladder cancer. ATRX [39], combined with MSI-H status has been shown to have high ICI treatment response.

However, the model identified some genes for which we couldn't find corresponding literature support. These include cancer-specific mutations, including *VHL* in renal cancer [40], *FGFR3*[41] in bladder cancer, *NF1* [42] in neural system cancer, *ESR1*[43] and *GATA3*[44], [45] in breast cancer. Although these genes have shown importance in that cancer type in general, we are unable to ascertain their function in immunotherapy.

**2. Attention maps for each type of cancer.**

In Figure 2a, we down-sampled the attention weight matrix for illustration purposes. Here we present the original attention maps (with sizes of 296 by 296). In the attention maps, the black areas represent low attention while the red or yellow areas represent high attention, indicating important gene interactions.

| **NSCLC**  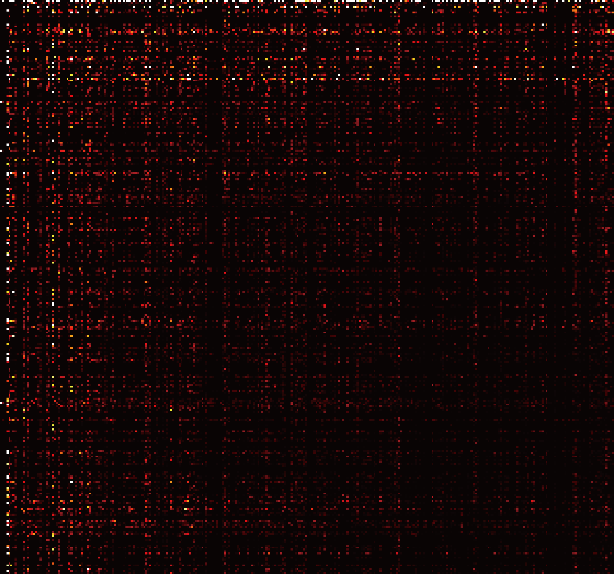 | **Melanoma**  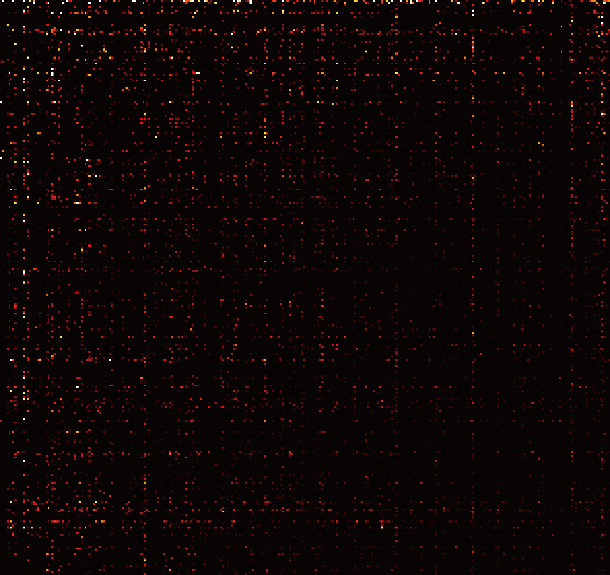 |
| --- | --- |
| **Bladder**  **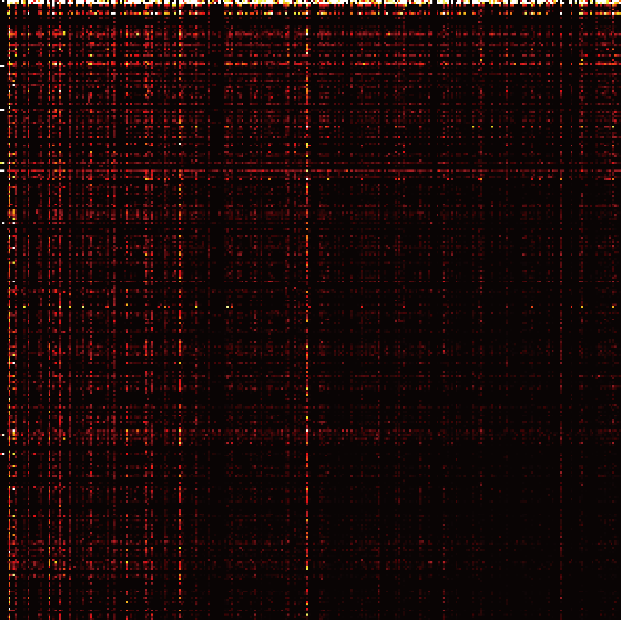** | **Renal Cell Carcinoma**  **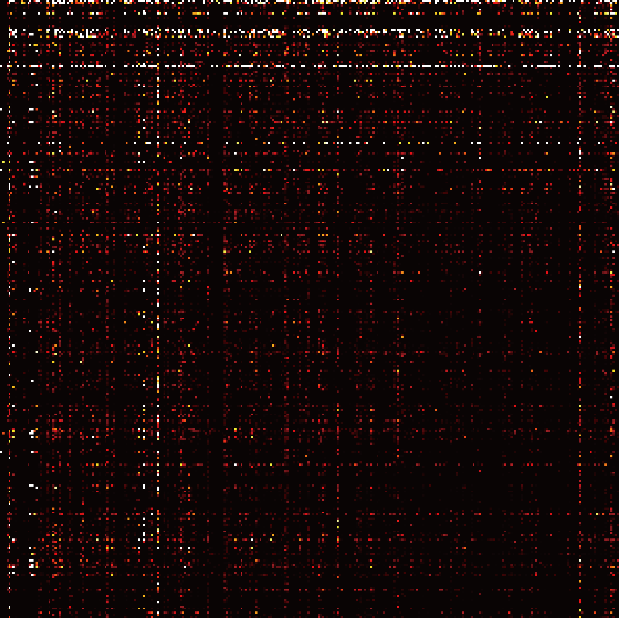** |

| **Head and Neck**  **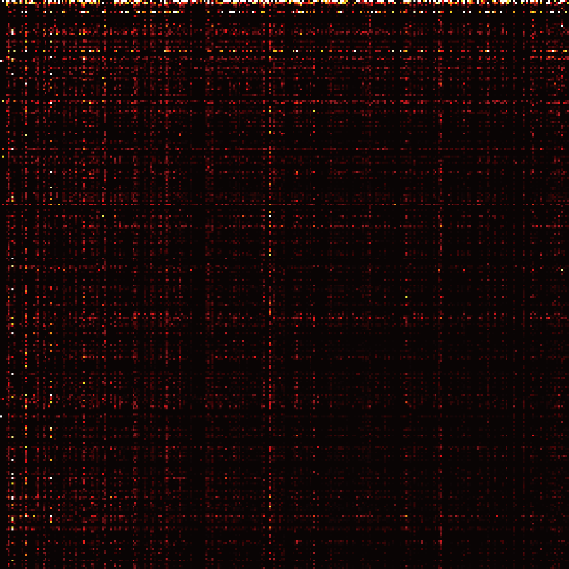** | **ESOPHAGOGASTRIC**  **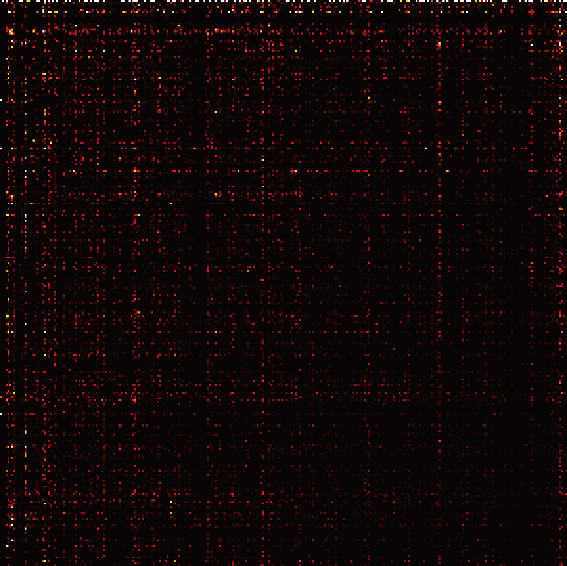** |
| --- | --- |
| **GLIOMA**  **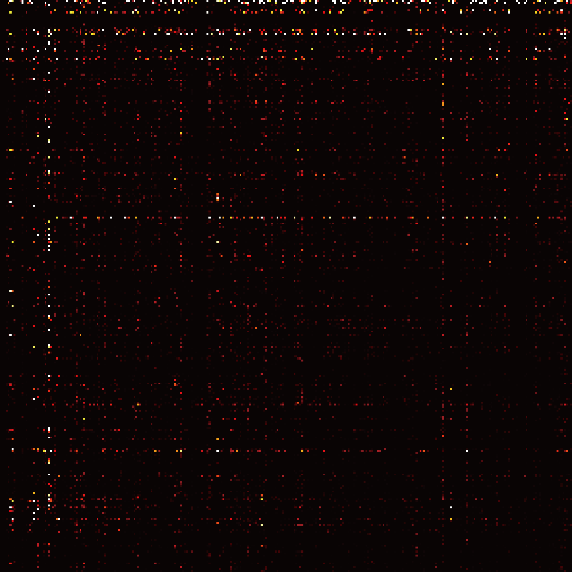** | **COLORECTAL**  **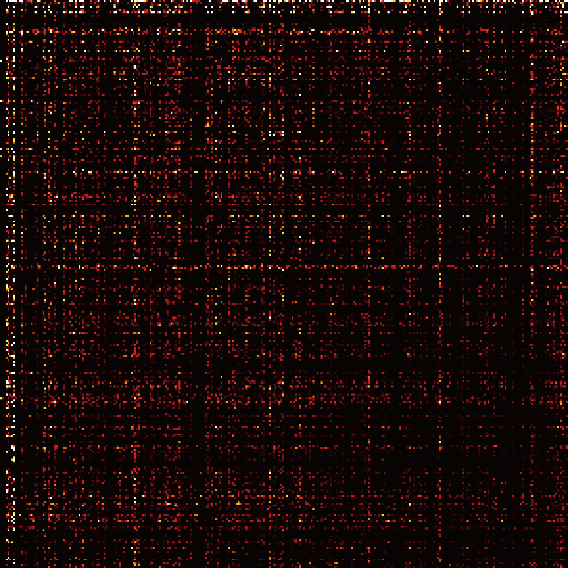** |
| **BREAST**  **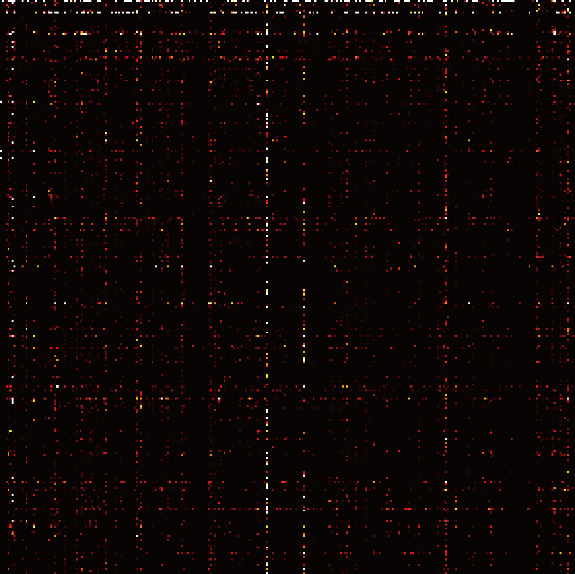** |  |
|  |  |

**3. Explore the embedding dimension.**

We investigate the effects of embedding dimension to model prediction accuracy (mean ± standard error). We conclude that except using a very low embedding dimension (e.g., five), which may lack necessary information to encode genes, keeping increasing the embedding sizes (i.e., 10, 50, 80) will not continue to improve the model accuracy.

| **Embed size** | **5** | **10** | **50** | **80** |
| --- | --- | --- | --- | --- |
| NSCLC | 0.567 ± 0.027 | 0.598 ± 0.016 | 0.601 ± 0.007 | 0.573 ± 0.026 |
| MELANOMA | 0.543 ± 0.017 | 0.575 ± 0.025 | 0.584 ± 0.013 | 0.585 ± 0.035 |
| BLADDER | 0.553 ± 0.032 | 0.574 ± 0.031 | 0.620 ± 0.016 | 0.600 ± 0.043 |
| RCC | 0.521 ± 0.044 | 0.610 ± 0.035 | 0.578 ± 0.024 | 0.575 ± 0.031 |
| HEAD & NECK | 0.544 ± 0.038 | 0.592 ± 0.024 | 0.610 ± 0.018 | 0.608 ± 0.039 |
| ESOPHAGO | 0.562 ± 0.038 | 0.569 ± 0.055 | 0.634 ± 0.022 | 0.610 ± 0.035 |
| GLIOMA | 0.461 ± 0.037 | 0.537 ± 0.032 | 0.534 ± 0.016 | 0.525 ± 0.037 |
| COLORECTAL | 0.453 ± 0.047 | 0.544 ± 0.033 | 0.556 ± 0.023 | 0.601 ± 0.047 |
| BREAST | 0.526 ± 0.061 | 0.582 ± 0.043 | 0.603 ± 0.023 | 0.617 ± 0.019 |

**4. Explore the weighting parameter k.**

We also investigate the calibrating results of *k_1_*, *k_2_* and *k_3_* across all cancer types. We obtain an average value of *k_1_* = 0.796 ± 0.0088, *k_2_* = 0.798 ± 0.0013, *k_3_* = 0.537 ± 0.35 across nine cancer types. *k_1_* (i.e., gene pathway length) and *k_2_* (i.e., gene centrality) have similar effects, while *k_3_* (i.e., penalizing the non-interacted gene pairs) have comparatively less influence on the attention weights.

**References**

[1] D. P. Lane, “p53, guardian of the genome,” *Nature*, vol. 358, no. 6381, Art. no. 6381, Jul. 1992, doi: 10.1038/358015a0.

[2] Y. Liu, P. L. Leslie, and Y. Zhang, “Life and Death Decision-Making by p53 and Implications for Cancer Immunotherapy,” *Trends Cancer*, vol. 7, no. 3, pp. 226–239, Mar. 2021, doi: 10.1016/j.trecan.2020.10.005.

[3] T. Jiang *et al.*, “Pan‐cancer analysis identifies TERT alterations as predictive biomarkers for immune checkpoint inhibitors treatment,” *Clin. Transl. Med.*, vol. 10, no. 2, p. e109, Jun. 2020, doi: 10.1002/ctm2.109.

[4] H. Li, J. Li, C. Zhang, C. Zhang, and H. Wang, “TERT mutations correlate with higher TMB value and unique tumor microenvironment and may be a potential biomarker for anti-CTLA4 treatment,” *Cancer Med.*, vol. 9, no. 19, pp. 7151–7160, 2020, doi: 10.1002/cam4.3376.

[5] T. Vidotto, C. M. Melo, E. Castelli, M. Koti, R. B. dos Reis, and J. A. Squire, “Emerging role of PTEN loss in evasion of the immune response to tumours,” *Br. J. Cancer*, vol. 122, no. 12, Art. no. 12, Jun. 2020, doi: 10.1038/s41416-020-0834-6.

[6] Z. Lin, L. Huang, S. L. Li, J. Gu, X. Cui, and Y. Zhou, “PTEN loss correlates with T cell exclusion across human cancers,” *BMC Cancer*, vol. 21, p. 429, Apr. 2021, doi: 10.1186/s12885-021-08114-x.

[7] S. Mafi *et al.*, “mTOR-Mediated Regulation of Immune Responses in Cancer and Tumor Microenvironment,” *Front. Immunol.*, vol. 12, 2022, Accessed: Feb. 13, 2023. [Online]. Available: https://www.frontiersin.org/articles/10.3389/fimmu.2021.774103

[8] M. A. Kelliher and J. E. Roderick, “NOTCH Signaling in T-Cell-Mediated Anti-Tumor Immunity and T-Cell-Based Immunotherapies,” *Front. Immunol.*, vol. 9, p. 1718, 2018, doi: 10.3389/fimmu.2018.01718.

[9] X. Li, X. Yan, Y. Wang, B. Kaur, H. Han, and J. Yu, “The Notch signaling pathway: a potential target for cancer immunotherapy,” *J. Hematol. Oncol.J Hematol Oncol*, vol. 16, no. 1, p. 45, May 2023, doi: 10.1186/s13045-023-01439-z.

[10] A. Allouch *et al.*, “CDKN1A is a target for phagocytosis-mediated cellular immunotherapy in acute leukemia,” *Nat. Commun.*, vol. 13, no. 1, Art. no. 1, Nov. 2022, doi: 10.1038/s41467-022-34548-3.

[11] J.-B. Cazier *et al.*, “Whole-genome sequencing of bladder cancers reveals somatic CDKN1A mutations and clinicopathological associations with mutation burden,” *Nat. Commun.*, vol. 5, no. 1, Art. no. 1, Apr. 2014, doi: 10.1038/ncomms4756.

[12] E. Adib *et al.*, “CDKN2A Alterations and Response to Immunotherapy in Solid Tumors,” *Clin. Cancer Res. Off. J. Am. Assoc. Cancer Res.*, vol. 27, no. 14, pp. 4025–4035, Jul. 2021, doi: 10.1158/1078-0432.CCR-21-0575.

[13] J. Liu *et al.*, “p300/CBP inhibition enhances the efficacy of programmed death-ligand 1 blockade treatment in prostate cancer,” *Oncogene*, vol. 39, no. 19, pp. 3939–3951, May 2020, doi: 10.1038/s41388-020-1270-z.

[14] M. Hu *et al.*, “ATM inhibition enhances cancer immunotherapy by promoting mtDNA leakage and cGAS/STING activation,” *J. Clin. Invest.*, vol. 131, no. 3, pp. e139333, 139333, Feb. 2021, doi: 10.1172/JCI139333.

[15] J. Li *et al.*, “Epigenetic driver mutations in ARID1A shape cancer immune phenotype and immunotherapy,” *J. Clin. Invest.*, vol. 130, no. 5, pp. 2712–2726, May 2020, doi: 10.1172/JCI134402.

[16] M. A. Coelho *et al.*, “Oncogenic RAS Signaling Promotes Tumor Immunoresistance by Stabilizing PD-L1 mRNA,” *Immunity*, vol. 47, no. 6, pp. 1083-1099.e6, Dec. 2017, doi: 10.1016/j.immuni.2017.11.016.

[17] D. R. Principe *et al.*, “Loss of SMAD4 Is Associated With Poor Tumor Immunogenicity and Reduced PD-L1 Expression in Pancreatic Cancer,” *Front. Oncol.*, vol. 12, 2022, Accessed: Feb. 13, 2023. [Online]. Available: https://www.frontiersin.org/articles/10.3389/fonc.2022.806963

[18] M. Lu *et al.*, “Pan-cancer analysis of SETD2 mutation and its association with the efficacy of immunotherapy,” *Npj Precis. Oncol.*, vol. 5, no. 1, Art. no. 1, Jun. 2021, doi: 10.1038/s41698-021-00193-0.

[19] Y. Li, Y. Song, J. Huang, and K. Wang, “APC mutations as a predictive marker of endometrial cancer immunotherapy: a retrospective cohort study,” *Lancet Oncol.*, vol. 23, p. S9, Jul. 2022, doi: 10.1016/S1470-2045(22)00408-9.

[20] H. Zhou *et al.*, “PBRM1 mutation and preliminary response to immune checkpoint blockade treatment in non-small cell lung cancer,” *Npj Precis. Oncol.*, vol. 4, no. 1, Art. no. 1, Mar. 2020, doi: 10.1038/s41698-020-0112-3.

[21] M. Chaker *et al.*, “Rho GTPase Effectors and NAD Metabolism in Cancer Immune Suppression,” *Expert Opin. Ther. Targets*, vol. 22, no. 1, pp. 9–17, Jan. 2018, doi: 10.1080/14728222.2018.1413091.

[22] D. Jeong *et al.*, “RhoA is associated with invasion and poor prognosis in colorectal cancer,” *Int. J. Oncol.*, vol. 48, no. 2, pp. 714–722, Feb. 2016, doi: 10.3892/ijo.2015.3281.

[23] R. Krupar *et al.*, “In silico analysis reveals EP300 as a panCancer inhibitor of anti-tumor immune response via metabolic modulation,” *Sci. Rep.*, vol. 10, no. 1, p. 9389, Jun. 2020, doi: 10.1038/s41598-020-66329-7.

[24] K. Takada *et al.*, “Exceptionally rapid response to pembrolizumab in a SMARCA4‐deficient thoracic sarcoma overexpressing PD‐L1: A case report,” *Thorac. Cancer*, vol. 10, no. 12, pp. 2312–2315, Dec. 2019, doi: 10.1111/1759-7714.13215.

[25] X. Chen, C. Su, S. Ren, C. Zhou, and T. Jiang, “Pan-cancer analysis of KEAP1 mutations as biomarkers for immunotherapy outcomes,” *Ann. Transl. Med.*, vol. 8, no. 4, p. 141, Feb. 2020, doi: 10.21037/atm.2019.11.52.

[26] J. Malhotra *et al.*, “Clinical outcomes and immune phenotypes associated with STK11 co-occurring mutations in non-small cell lung cancer,” *J. Thorac. Dis.*, vol. 14, no. 6, pp. 1772–1783, Jun. 2022, doi: 10.21037/jtd-21-1377.

[27] R. Pinyol, D. Sia, and J. M. Llovet, “Immune Exclusion-Wnt/CTNNB1 Class Predicts Resistance to Immunotherapies in HCC,” *Clin. Cancer Res.*, vol. 25, no. 7, pp. 2021–2023, Apr. 2019, doi: 10.1158/1078-0432.CCR-18-3778.

[28] E. S. Knudsen, S. C. Pruitt, P. A. Hershberger, A. K. Witkiewicz, and D. W. Goodrich, “Cell cycle and beyond: Exploiting new RB1 controlled mechanisms for cancer therapy,” *Trends Cancer*, vol. 5, no. 5, pp. 308–324, May 2019, doi: 10.1016/j.trecan.2019.03.005.

[29] R. G. Manzano, A. Catalan-Latorre, and A. Brugarolas, “RB1 and TP53 co-mutations correlate strongly with genomic biomarkers of response to immunity checkpoint inhibitors in urothelial bladder cancer,” *BMC Cancer*, vol. 21, no. 1, p. 432, Apr. 2021, doi: 10.1186/s12885-021-08078-y.

[30] C. Shi, Y. Wang, J. Xue, and X. Zhou, “Immunotherapy for EGFR-mutant advanced non-small-cell lung cancer: Current status, possible mechanisms and application prospects,” *Front. Immunol.*, vol. 13, 2022, Accessed: Jul. 05, 2023. [Online]. Available: https://www.frontiersin.org/articles/10.3389/fimmu.2022.940288

[31] K. K. W. To, W. Fong, and W. C. S. Cho, “Immunotherapy in Treating EGFR-Mutant Lung Cancer: Current Challenges and New Strategies,” *Front. Oncol.*, vol. 11, 2021, Accessed: Jul. 05, 2023. [Online]. Available: https://www.frontiersin.org/articles/10.3389/fonc.2021.635007

[32] H. Zeng *et al.*, “The Predictive Value of PAK7 Mutation for Immune Checkpoint Inhibitors Therapy in Non-Small Cell Cancer,” *Front. Immunol.*, vol. 13, p. 834142, 2022, doi: 10.3389/fimmu.2022.834142.

[33] P. A. Ascierto *et al.*, “The role of BRAF V600 mutation in melanoma,” *J. Transl. Med.*, vol. 10, p. 85, Jul. 2012, doi: 10.1186/1479-5876-10-85.

[34] S. Naderi-Azad and R. Sullivan, “The potential for BRAF-targeted therapy combined with immunotherapy in melanoma,” *Expert Rev. Anticancer Ther.*, vol. 20, no. 2, pp. 131–136, Feb. 2020, doi: 10.1080/14737140.2020.1724097.

[35] Z. Wang *et al.*, “Combination of AKT1 and CDH1 mutations predicts primary resistance to immunotherapy in dMMR/MSI-H gastrointestinal cancer,” *J. Immunother. Cancer*, vol. 10, no. 6, p. e004703, Jun. 2022, doi: 10.1136/jitc-2022-004703.

[36] J. Zou *et al.*, “Casp8 acts through A20 to inhibit PD-L1 expression: The mechanism and its implication in immunotherapy,” *Cancer Sci.*, vol. 112, no. 7, pp. 2664–2678, Jul. 2021, doi: 10.1111/cas.14932.

[37] C. R. Figueiredo *et al.*, “Loss of BAP1 expression is associated with an immunosuppressive microenvironment in uveal melanoma, with implications for immunotherapy development,” *J. Pathol.*, vol. 250, no. 4, pp. 420–439, 2020, doi: 10.1002/path.5384.

[38] X. Chen, X. Lin, G. Pang, J. Deng, Q. Xie, and Z. Zhang, “Significance of KDM6A mutation in bladder cancer immune escape,” *BMC Cancer*, vol. 21, no. 1, p. 635, May 2021, doi: 10.1186/s12885-021-08372-9.

[39] Y. Ge, Z. Wang, H. Li, Y. Liu, and P. Wei, “Association of ATRX mutations with immunologically active characteristics in patients with MSI-prone tumors,” *Am. J. Transl. Res.*, vol. 14, no. 9, pp. 6107–6122, Sep. 2022.

[40] E. Kim and S. Zschiedrich, “Renal Cell Carcinoma in von Hippel–Lindau Disease—From Tumor Genetics to Novel Therapeutic Strategies,” *Front. Pediatr.*, vol. 6, p. 16, Feb. 2018, doi: 10.3389/fped.2018.00016.

[41] A. Kacew and R. F. Sweis, “FGFR3 Alterations in the Era of Immunotherapy for Urothelial Bladder Cancer,” *Front. Immunol.*, vol. 11, p. 575258, Nov. 2020, doi: 10.3389/fimmu.2020.575258.

[42] K. B. Haworth *et al.*, “Immune profiling of NF1-associated tumors reveals histologic subtype distinctions and heterogeneity: implications for immunotherapy,” *Oncotarget*, vol. 8, no. 47, pp. 82037–82048, May 2017, doi: 10.18632/oncotarget.18301.

[43] J. O. Brett, L. M. Spring, A. Bardia, and S. A. Wander, “ESR1 mutation as an emerging clinical biomarker in metastatic hormone receptor-positive breast cancer,” *Breast Cancer Res.*, vol. 23, no. 1, p. 85, Aug. 2021, doi: 10.1186/s13058-021-01462-3.

[44] N. K. Yoon *et al.*, “Higher Levels of GATA3 Predict Better Survival in Women with Breast Cancer,” *Hum. Pathol.*, vol. 41, no. 12, pp. 1794–1801, Dec. 2010, doi: 10.1016/j.humpath.2010.06.010.

[45] Q. Zhang *et al.*, “GATA3 Predicts the Tumor Microenvironment Phenotypes and Molecular Subtypes for Bladder Carcinoma,” *Front. Surg.*, vol. 9, p. 860663, May 2022, doi: 10.3389/fsurg.2022.860663.
