# Supplementary material for: Biology-aware mutation-based deep learning for outcome prediction of cancer immunotherapy with immune checkpoint inhibitors
Source: NPJ Precis Oncol. 2023 Nov 6;7:117. doi: 10.1038/s41698-023-00468-8 (PMC10628135; doi:10.1038/s41698-023-00468-8)
Supplement: Supplementary file 2 — REPORTING SUMMARY [file 41698_2023_468_MOESM2_ESM.pdf]

nature portfolio

Corresponding author(s): Lei Xing
Last updated by author(s): 2023/8/9

Reporting Summary

Nature Portfolio wishes to improve the reproducibility of the work that we publish. This form provides structure for consistency and transparency in reporting. For further information on Nature Portfolio policies, see our Editorial Policies and the Editorial Policy Checklist.

Statistics

For all statistical analyses, confirm that the following items are present in the figure legend, table legend, main text, or Methods section.

n/a	Confirmed	
☐	☒	The exact sample size (*n*) for each experimental group/condition, given as a discrete number and unit of measurement
☒	☐	A statement on whether measurements were taken from distinct samples or whether the same sample was measured repeatedly
☐	☒	The statistical test(s) used AND whether they are one- or two-sided<br>*Only common tests should be described solely by name; describe more complex techniques in the Methods section.*
☒	☐	A description of all covariates tested
☐	☒	A description of any assumptions or corrections, such as tests of normality and adjustment for multiple comparisons
☐	☒	A full description of the statistical parameters including central tendency (e.g. means) or other basic estimates (e.g. regression coefficient) AND variation (e.g. standard deviation) or associated estimates of uncertainty (e.g. confidence intervals)
☐	☒	For null hypothesis testing, the test statistic (e.g. *F*, *t*, *r*) with confidence intervals, effect sizes, degrees of freedom and *P* value noted<br>*Give P values as exact values whenever suitable.*
☒	☐	For Bayesian analysis, information on the choice of priors and Markov chain Monte Carlo settings
☒	☐	For hierarchical and complex designs, identification of the appropriate level for tests and full reporting of outcomes
☒	☐	Estimates of effect sizes (e.g. Cohen's *d*, Pearson's *r*), indicating how they were calculated

Our web collection on statistics for biologists contains articles on many of the points above.

Software and code

Policy information about availability of computer code

Data collection	No software was used.
Data analysis	The code used for model analysis are accessible from https://github.com/JunyanReplicant/ICImodel

For manuscripts utilizing custom algorithms or software that are central to the research but not yet described in published literature, software must be made available to editors and reviewers. We strongly encourage code deposition in a community repository (e.g. GitHub). See the Nature Portfolio guidelines for submitting code & software for further information.

Data

Policy information about availability of data

All manuscripts must include a data availability statement. This statement should provide the following information, where applicable:

- Accession codes, unique identifiers, or web links for publicly available datasets
- A description of any restrictions on data availability
- For clinical datasets or third party data, please ensure that the statement adheres to our policy

All data used for analysis are accessible from public database cBioPortal (https://www.cbioportal.org/).

Research involving human participants, their data, or biological material

Policy information about studies with human participants or human data. See also policy information about sex, gender (identity/presentation), and sexual orientation and race, ethnicity and racism.

Reporting on sex and gender	Sex and gender is not relevant in this study, and sex and gender information is not available in our dataset. The data are processed equally regardless of sex and gender.
Reporting on race, ethnicity, or other socially relevant groupings	Race, ethnicity or other socially groupings are not relevant in this study, and this information is not available in our dataset. The data are processed equally regardless of race, ethnicity, or other socially groupings.
Population characteristics	All patients are diagnosed with cancer, treated with the same type of immunotherapy (Immune Checkpoint Inhibitors), and have gene mutation profile.
Recruitment	N/A. We use a public dataset that has been institutionally reviewed and approved by MSKCC and downloaded from public database cBioPortal. No selection bias or other bias exist in the study.
Ethics oversight	Memorial Sloan Kettering Cancer Center

Note that full information on the approval of the study protocol must also be provided in the manuscript.

Field-specific reporting

Please select the one below that is the best fit for your research. If you are not sure, read the appropriate sections before making your selection.

☒ Life sciences ☐ Behavioural & social sciences ☐ Ecological, evolutionary & environmental sciences

For a reference copy of the document with all sections, see nature.com/documents/nr-reporting-summary-flat.pdf

Life sciences study design

All studies must disclose on these points even when the disclosure is negative.

Sample size	1571
Data exclusions	Patients whose cancer types are labeled unknown origin are removed.
Replication	The model findings are repeated and verified on 9 types of cancer, which includes includes 349 NSCLC, 320 melanoma, 215 bladder cancer, 151 RCC, 139 head and neck, 126 esophagogastric cancer, 117 glioma, 110 colorectal cancer, and 44 breast cancer patients.
Randomization	We use a public dataset from Memorial Sloan Kettering Cancer Center and downloaded from public database cBioPortal. In the original study, the dataset are randomized during the experiments.
Blinding	For each type of cancer, we randomly split the training, validation, and test set during model training and validation, resulting in a ten-fold cross validation scheme. Thus the investigators are blinding to the patients allocation during our model analysis.

Reporting for specific materials, systems and methods

We require information from authors about some types of materials, experimental systems and methods used in many studies. Here, indicate whether each material, system or method listed is relevant to your study. If you are not sure if a list item applies to your research, read the appropriate section before selecting a response.

Materials & experimental systems

n/a	Involved in the study	
☒	☐	Antibodies
☒	☐	Eukaryotic cell lines
☒	☐	Palaeontology and archaeology
☒	☐	Animals and other organisms
☒	☐	Clinical data
☒	☐	Dual use research of concern
☒	☐	Plants

Methods

n/a	Involved in the study	
☒	☐	ChIP-seq
☒	☐	Flow cytometry
☒	☐	MRI-based neuroimaging
